# Fatal Intoxication of European Yew (*Taxus baccata* L.) in Two Donkeys in North-Eastern Italy: A Case Report

**DOI:** 10.3390/toxics14040294

**Published:** 2026-03-28

**Authors:** Luca Spadotto, Silva Rubini, Laura Cornara, Federica Betuzzi, Mariarosaria Ingegneri, Antonella Smeriglio, Domenico Trombetta, Cinzia Centelleghe, Sandro Mazzariol

**Affiliations:** 1 Department of Comparative Biomedicine and Food Science (BCA), University of Padova, Viale Dell’Università 16, 35020 Legnaro, PD, Italy; luca.spadotto@studenti.unipd.it (L.S.); sandro.mazzariol@unipd.it (S.M.); 2 Istituto Zooprofilattico Sperimentale Della Lombardia e Dell’Emilia-Romagna (IZSLER), Via Modena 483, 44044 Cassana, FE, Italy; silva.rubini@izsler.it; 3 Department of Earth, Environmental and Life Sciences (DISTAV), University of Genova, Corso Europa 26, 16132 Genova, GE, Italy; laura.cornara@unige.it (L.C.); federica.betuzzi@edu.unige.it (F.B.); 4 Department of Chemical, Biological, Pharmaceutical and Environmental Sciences (CHIBIOFARAM), University of Messina, Via Ferdinando Stagno D’Alcontres 31, 98166 Messina, ME, Italy; mariarosaria.ingegneri@unime.it (M.I.); antonella.smeriglio@unime.it (A.S.); domenico.trombetta@unime.it (D.T.)

**Keywords:** plant poisoning, herbivorous mammals, diagnostic pathology, botanical identification, LC-ESI-MS, taxane alkaloids, cardiotoxicity, gastric content

## Abstract

Poisoning caused by *Taxus baccata* is a well-known cause of sudden death in domestic animals due to the cardiotoxic effects of taxine alkaloids. This study describes two cases of fatal intoxication in donkeys (*Equus africanus asinus*) and demonstrates a multidisciplinary diagnostic approach combining pathology, botanical identification, and toxicology. Two animals were found dead without prior clinical signs on a farm in north-eastern Italy. Necropsies were performed, and samples were collected for further investigations. Histopathological findings were limited and non-specific, consistent with the hyperacute course typical of yew poisoning. Fragments of plant material resembling yew needles and twigs were identified in the gastric contents. Toxicological analysis using liquid chromatography–electrospray ionization mass spectrometry confirmed the presence of taxane alkaloids, supporting the diagnosis of yew poisoning. These data highlight the importance of integrating necropsy results with botanical examination and targeted toxicological analyses in cases of suspected plant poisoning. This multidisciplinary approach provides a reliable diagnostic framework for confirming yew poisoning in veterinary investigations.

## 1. Introduction

Poisoning from toxic plants represents a relevant cause of morbidity and sudden death in domestic animals, particularly under conditions of limited feed availability or accidental ingestion. Among toxic plants, species belonging to the genus *Taxus* are of particular concern due to their high cardiotoxic potential.

European yew (*Taxus baccata* L.) is an evergreen coniferous tree belonging to the *Taxaceae* family, capable of reaching up to 28 m in height. This species is native to the European continent, the Atlas Mountains in northern Africa, and Asia Minor [1]. In Italy, it is widely distributed, although considered locally rare in some regions [2]. Due to its slow growth and dense foliage, it is primarily grown as an ornamental plant with many uses in landscaping [3]. All parts of the plant are toxic, except for the fleshy red aril surrounding the seed [4].

Yew possesses a variety of toxic compounds, including alkaloids, taxane derivatives, and glycosides. Among these, the most toxic constituents are taxine alkaloids, primarily taxine A and taxine B, together with related compounds such as isotaxine B, paclitaxel (taxol A), and taxol B [5,6]. Taxine B represents the major fraction of the total alkaloid content and is considered the principal contributor to toxicity [6].

The toxic effects of yew are mainly due to the action of taxine alkaloids on the cardiovascular system [7]. Taxine B interferes with cardiac impulse conduction by blocking calcium and sodium channels in cardiomyocytes, resulting in severe conduction disturbances and myocardial depression [7,8]. This mechanism explains the rapid onset of clinical signs, including bradycardia, arrhythmias, atrioventricular block, and ultimately cardiac arrest. Furthermore, in a comparative study, the inhibitory effects of taxine on the peristaltic movements of the gastrointestinal tract were examined [9]. The findings suggested that yew effects on intestinal functions are likely not caused by taxine, but are rather the result of other plant constituents, likely belonging to non-alkaloid fractions (e.g., lipophilic components) [9]. Following absorption in the gastrointestinal tract, taxine alkaloids are rapidly distributed to highly perfused organs, particularly the heart, where they exert their toxic effects. Taxoids are primarily metabolized in the liver by cytochrome P450 enzymes (mainly CYP2C8 and CYP3A4) and eliminated mainly via biliary excretion, with only a small fraction excreted in urine [10,11,12].

For both animals and humans, toxic effects are usually rapid, due to the rapid absorption in the digestive tract. Clinical manifestations are initially nonspecific but may rapidly progress to severe cardiovascular impairment including bradycardia, hypotension, ventricular arrhythmias, atrioventricular block and cardiac arrest [12,13,14,15].

Even though *Taxus* intoxications are statistically rare, various cases of human and animal poisoning have been described in the literature [16,17,18,19,20]. Most human cases are associated with intentional ingestion or suicide attempts, whereas poisoning in animals typically occurs accidentally, as *T. baccata* is generally unpalatable. However, ingestion may still occur under particular conditions, such as limited availability of alternative feed (e.g., during winter) or accidental mixing with more palatable forage [21]. In addition, *Taxus* species and other poisonous trees or shrubs are often planted around fences and barns, as cattle owners are unaware of their toxic potential [22]. Reported lethal doses vary among species, but ingestion of small amounts of plant material may be sufficient to cause sudden death in equids [23].

Diagnosis of yew poisoning can be challenging, as clinical signs are often absent or nonspecific and death may occur rapidly. Therefore, a multidisciplinary approach integrating pathological findings, botanical identification, and toxicological analysis is essential for a reliable diagnosis.

The aim of this study was to describe fatal intoxication by European yew in two donkeys (*Equus africanus asinus*) and to highlight the value of an integrated diagnostic approach combining pathological, botanical, and toxicological investigations.

## 2. Case Presentation and Analytical Procedures

### 2.1. Case History and Anatomopathological Examination

Two donkeys (*E. africanus asinus*), raised in the same farm in mainland Venice (north-eastern Italy), with no previous clinical signs, were found dead on 20 December 2023. The owners reported a small amount of thin leaves and sticks compatible with *T. baccata* within the paddock where the animals were found. Upon further inspection, the presence of a yew tree near the grazing area was confirmed. Additional details are provided in Supplementary Figure S1a,b.

The animals were transported the following day to the Department of Comparative Biomedicine and Food Science, University of Padua, for post-mortem examination. The two subjects were an 8-year-old female (111 kg; case 1) and a 15-year-old female (133 kg; case 2). A complete and standardized necropsy was performed by veterinary pathologists. Both animals were in good nutritional condition.

During necroscopy, major organs (liver, lungs, heart, stomach, intestine, spleen, and kidneys) were collected for histopathological examination and fixed in 10% neutral buffered formalin. Samples were subsequently dehydrated and embedded in paraffin following standard procedures. Sections (3–5 μm) of formalin-fixed, paraffin-embedded (FFPE) tissues were obtained, mounted on glass slides, and routinely stained with hematoxylin and eosin.

Based on clinical history and suspicion of *T. baccata* ingestion, gastric contents were collected. In addition, gastric content, feces, liver, and kidney samples were individually stored in plastic vials at −20 °C for toxicological analysis.

### 2.2. Botanical Analysis

Plant material recovered from the gastric contents was submitted to the Department of Earth, Environmental and Life Sciences (DISTAV), University of Genoa, for botanical identification. Prior to analysis, samples were rinsed in 70% ethanol for 24 h [24]. Samples were examined using a LEICA M205 C stereomicroscope (Leica Microsystems, Wetzlar, Germany) to identify plant residues. Portions of leaves and twigs, morphologically consistent with gymnosperms, were compared with reference material of *T. baccata* obtained from the Botanical Garden of the University of Genoa (Italy). Micromorphological and anatomical features of both plant fragments and reference material were analyzed after manual sectioning with double-edged razor blades. Leaf and twigs sections, as well as leaf epidermal peels, were processed as follows: (i) mounted in water and directly observed; (ii) cleared with an aqueous chloral hydrate solution and mounted in a chloral hydrate–glycerol solution to prevent crystallization [25]; (iii) cleared and stained with phloroglucinol-HCl (Merck, Darmstadt, Germany) for lignin detection. Observations were performed using a Leica DM 2000 transmission light microscope equipped with a DFC 320 camera (Leica Microsystems, Wetzlar, Germany).

Samples of gastric contents and reference plant material were fixed for 24 h in FineFIX working solution (Milestone Srl, Sorisole, Bergamo, Italy) at 4 °C [26], dehydrated through a graded ethanol series, and dried using liquid CO_2_ in a critical point dryer (K850CPD 2M, Strumenti S.r.l., Rome, Italy).

Samples were mounted on aluminum stubs using carbon adhesive tape and sputter-coated with a 10 nm gold layer. Analyses were carried out using a Tescan LMU SEM VEGA3 microscope (Tescan USA Inc., Cranberry Twp, PA, USA) operating at an accelerating voltage of 20 kV.

### 2.3. LC-ESI-MS Analysis

Approximately 0.5 g of each post-mortem sample (gastric content, liver, kidney, and feces) was mixed with 2 mL of 0.01 M sodium carbonate buffer (pH 9.5) according to Frommherz et al., with minor modifications [27]. The mixture was homogenized and centrifuged at 3500× *g* for 20 min. The supernatant was loaded onto an activated Bond Elut solid-phase extraction (SPE) cartridge (Agilent Technologies Inc., Santa Clara, CA, USA), previously conditioned with 1 mL methanol, 1 mL water, and 1 mL of 0.01 M sodium carbonate buffer (pH 9.5). After sample loading, the cartridge was washed with 3 mL of the same buffer and dried under vacuum for 3 min. Analytes were eluted with two aliquots of 0.7 mL methanol. Eluates were combined and evaporated to dryness under a gentle nitrogen stream at room temperature, then reconstituted in 1 mL methanol. All samples were filtered through a 0.20 μm nylon syringe filter (Captiva, Agilent Technologies Inc.) prior to LC-ESI-MS analysis. Chromatographic analysis was performed using an Agilent 1200 HPLC system equipped with a degasser (G1379B), binary pump (G1312A), autosampler (G1329A), and column oven (G1330B), coupled to a 6320-ion trap mass spectrometer (G2446A). Separation was achieved on a Luna Omega PS C18 column (150 × 2.1 mm, 5 μm; Phenomenex, Torrance, CA, USA) at 35 °C using a mobile phase consisting of water with 0.1% formic acid (A) and acetonitrile with 0.1% formic acid (B), with the following gradient: 80% A (0–0.5 min), 80–40% A (0.5–7 min), 40–20% A (7–12 min), 20–5% A (12–15 min), 5–0% A (15–16 min), 0% A (16–17 min), 0–80% A (17–18 min), and 80% A (18–22 min). The flow rate was 0.4 mL/min. The injection volume was 5 μL, and samples were maintained at 4 °C in the autosampler. The mass spectrometer operated in positive ionization mode (ESI+) under the following conditions: capillary voltage 3.5 kV, nebulizer pressure 40 psi (N_2_), drying gas temperature 350 °C, drying gas flow 12 L/min, and skimmer voltage 40 V. Data were acquired in full-scan mode over the *m*/*z* range 90–2000.

Data acquisition was performed using Agilent ChemStation software (version B.01.03) and Trap Control software (version 6.2).

Due to the limited availability of authentic reference standards for taxine derivatives, only selected compounds (paclitaxel and 10-deacetylbaccatin III) were confirmed using standards, while the remaining analytes were tentatively assigned based on retention time, observed [M + H]^+^ ions, and comparison with literature data. In cases where multiple compounds exhibited identical [M + H]^+^ ions, they were differentiated based on chromatographic separation and reported as distinct isomeric forms.

## 3. Results

**Figure 1:**
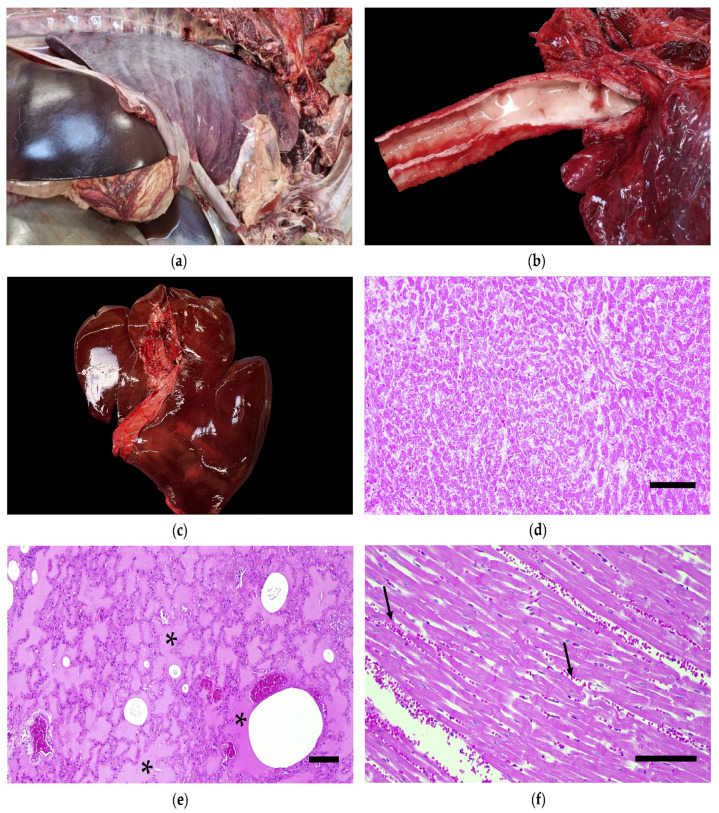
Macroscopic (**a**–**c**) and histological (**d**–**f**) examination of organs and tissues from donkey (case 2) following fatal *Taxus baccata* intoxication. (**a**) General view of the open abdominal and thoracic cavities. (**b**) Detail of the tracheal lumen showing abundant foamy content. (**c**) Liver; the organ appeared enlarged and pale. (**d**) Liver showing severe multifocal to coalescing hepatic necrosis, scale bar = 100 μm. (**e**) Lung showing diffuse pulmonary edema (asterisk) and congestion; scale bar = 100 μm. (**f**) Heart showing multifocal myocardial hemorrhages (arrows); scale bar = 50 μm. Tissues were stained with hematoxilin and eosin (**d**–**f**).

At gross examination, the oral, ocular and genital mucosae were pale. Mild serosanguineous effusion within pericardial, thoracic, and abdominal cavities (Figure 1a) was noted, along with severe, diffuse pulmonary edema, characterized by abundant foam in the tracheal lumen (Figure 1b), and petechiae on the renal parenchyma. The livers of both animals were enlarged with diffusely pale and softened parenchyma (Figure 1c). A small amount (approximately 5 g) of flat, needle-shaped green leaves was found in the gastric lumen of case 1, whereas in case 2, the plant material was not macroscopically identifiable. The hearts of both animals exhibited morphological alterations, with marked right ventricular dilation.

Histological examination revealed multifocal areas of hepatic necrosis (Figure 1d), along with multifocal lymphocytic inflammatory infiltrates, occasionally associated with neutrophils. Additionally, hepatocellular degeneration in non-necrotic areas and a slight increase in the fibrous component within the portal spaces were observed. In the intestinal mucosa and submucosa, a diffuse, moderate lymphocytic and eosinophilic inflammatory infiltrate was observed. Microscopic examination confirmed severe pulmonary edema and congestion, with mild multifocal intra-alveolar hemorrhages (Figure 1e), along with interstitial lymphocytic inflammatory infiltration ranging from multifocal to coalescing patterns. Finally, mild to moderate, multifocal to coalescing hemorrhagic foci were observed in the myocardium in one animal (Figure 1f).

Stereomicroscopic observation of fecal material from both donkeys did not allow the identification of recognizable plant fragments, likely due to the advanced degradation of the samples.

**Figure 2:**
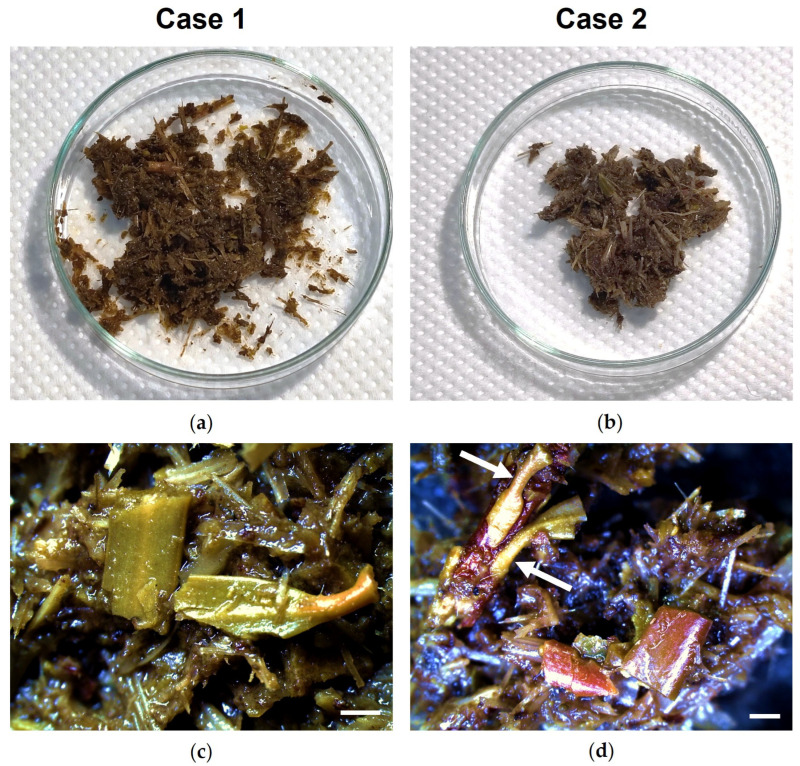
Stereomicroscope analysis of the gastric content collected from two donkeys (case 1 and 2) following fatal *Taxus baccata* intoxication. (**a**,**b**) General view of the gastric content. (**c**) Central and basal portions of partially digested gymnosperm needles. (**d**) Partially digested twigs, with visible needle petioles (arrows). Scale bars = 1 mm. (**c**) 1.25×; (**d**) 1×.

In contrast, stereomicroscopic analysis of the gastric content from both animals allowed the identification of partially digested plant fragments resembling gymnosperm needles and twigs (Figure 2a,b). At higher magnification, the gymnosperm fragments found in the stomach content of case l appeared as different portions of partially digested needles (Figure 2c), whereas those found in case 2 were mainly represented by small, fragmented twigs (Figure 2d).

**Figure 3:**
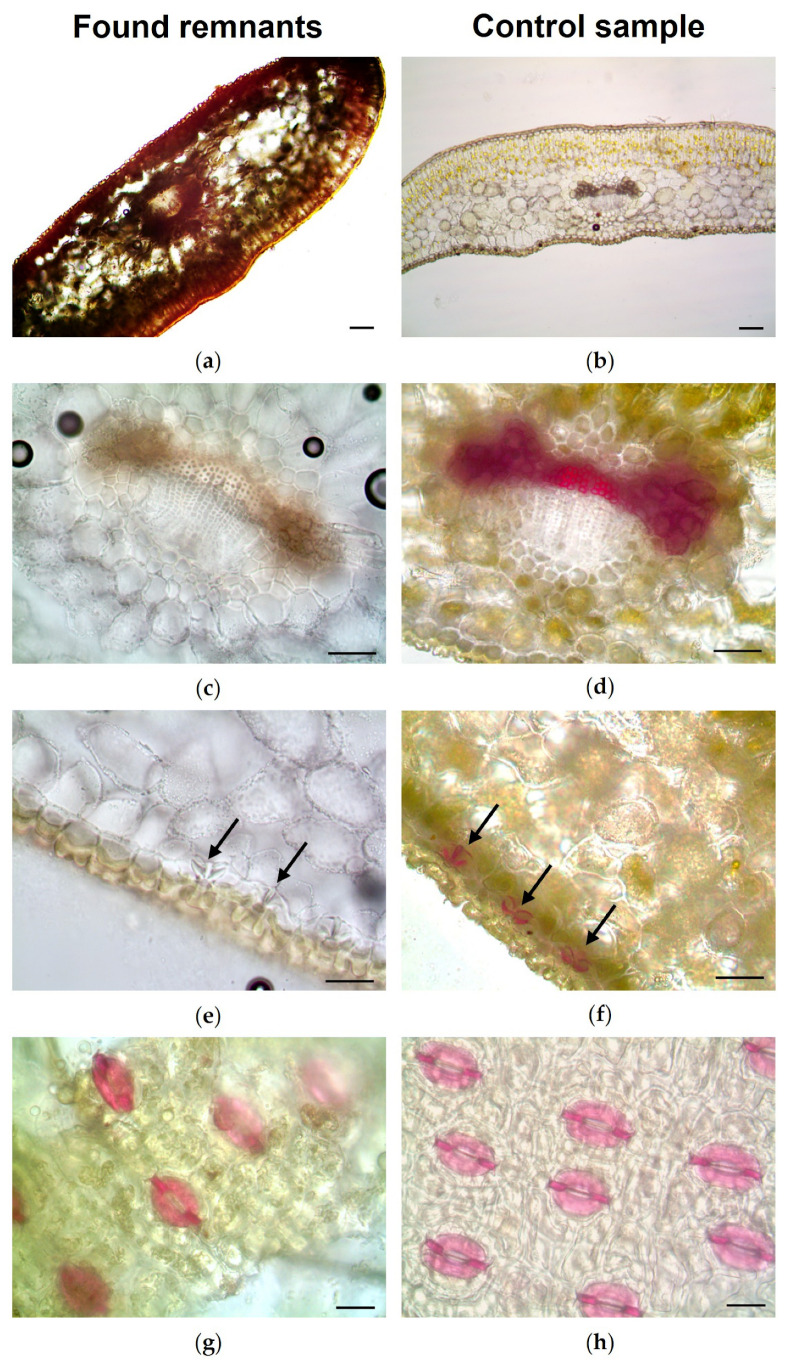
Light microscopy of remnants of needles found in gastric content collected from two donkeys (case 1 and 2) following fatal *Taxus baccata* intoxication (**a**,**c**,**e**,**g**) and reference *T. baccata* needles (**b**,**d**,**f**,**h**). (**b**,**c**,**e**) Chloral hydrate; (**d**,**f**–**h**) Phloroglucinol-HCl. (**a**–**f**) Transverse section. (**c**,**d**) Detail of the single central vascular bundle surrounded by transfusion tissue. (**e**,**f**) Papillose epidermis and sunken stomata (arrows) on the abaxial surface. (**g**,**h**) Paradermal view showing stomata. Scale bars = 100 μm (**a**,**b**); 50 μm (**c**–**f**); 25 μm (**g**,**h**).

**Figure 4:**
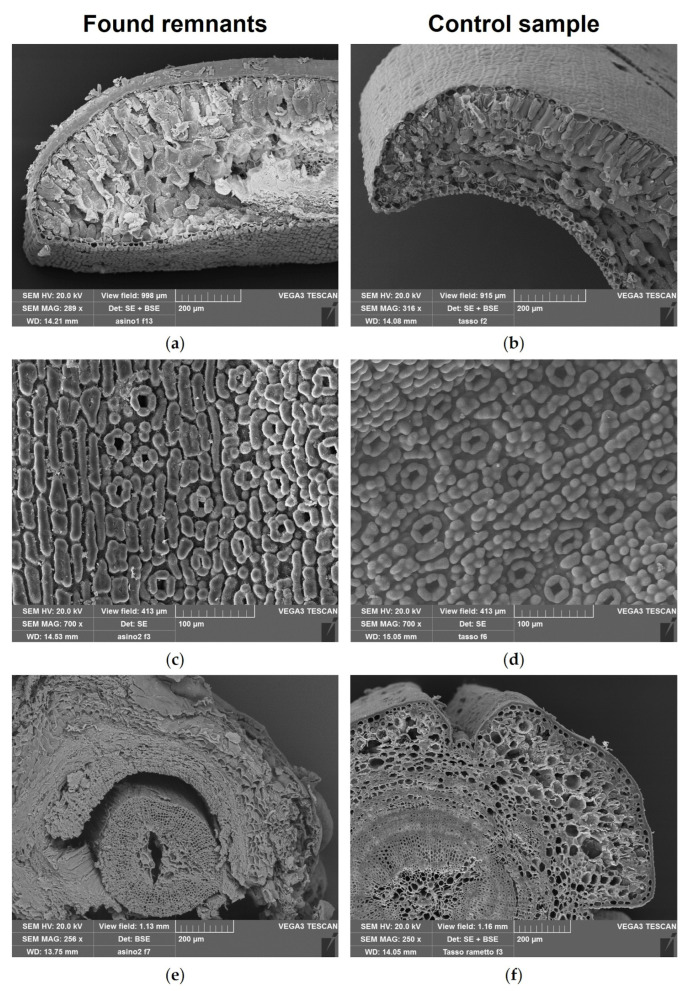
SEM analysis of remnants found in gastric content collected from two donkeys (case 1 and 2) following fatal *Taxus baccata* intoxication (**a**,**c**,**e**) and reference *T. baccata* needle and twig samples (**b**,**d**,**f**). (**a**,**b**) Needle transverse section. (**c**,**d**) Needle abaxial surface showing stomata. (**e**,**f**) Twig transverse section.

**Figure 5:**
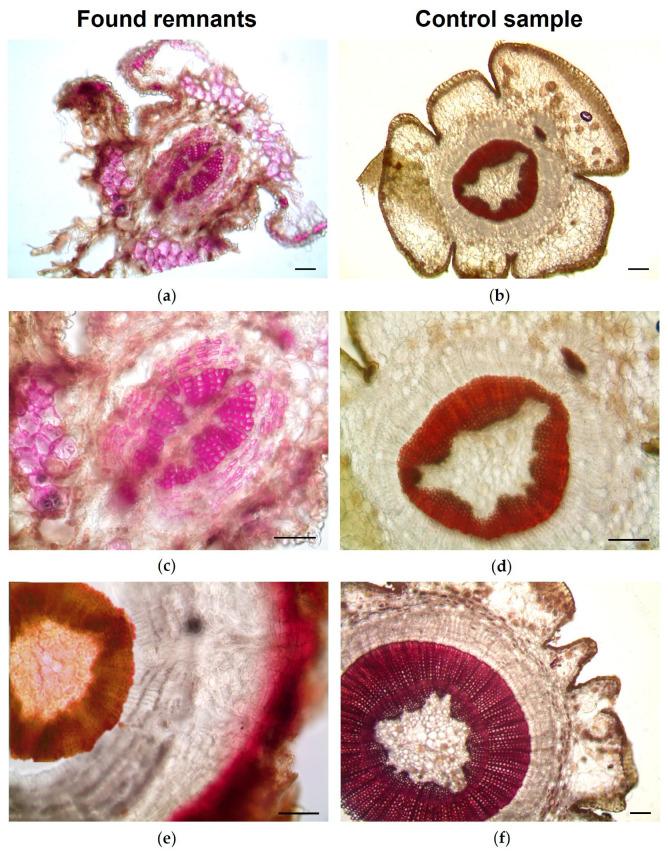
Light microscopy of twig remnants found in gastric content collected from two donkeys (case 1 and 2) after fatal *Taxus baccata* intoxication (**a**,**c**,**e**) and reference *T. baccata* twig samples (**b**,**d**,**f**). (**a**–**f**) Transverse sections stained with Phloroglucinol-HCl. (**a**,**b**) General view. (**c**–**f**) Detail of the central ring composed of homoxylous wood (**c**,**d**) and of the cortical zone (**e**,**f**). Scale bars = 100 μm.

Light microscopy and scanning electron microscopy of sections and/or peels of these fragments, compared with those obtained from needles and twigs of the reference material (Figure 3, Figure 4 and Figure 5), confirmed the presence of *T. baccata* in the gastric contents of both animals.

The main diagnostic features of the needles included: (i) a bilayered mesophyll (Figure 3a,b and Figure 4a,b), with a single vascular bundle surrounded by a distinct endodermis and transfusion tissue present on both sides (Figure 3c,d); (ii) stomata restricted to the abaxial surface and sunken below papillose epidermal cells (Figure 3e,f); (iii) stomata arranged in longitudinal rows and characterized by short, thin polar hooks (Figure 3g,h) and subsidiary cells located above the upper lamellae (Figure 4c,d); (iv) papilliform epidermal cells fused and arranged in block-like patterns between stomata (Figure 4c,d). In addition, histochemical analysis demonstrated lignification of stomatal subsidiary cells (Figure 3f–h).

The main diagnostic features of the twigs included: (i) an irregular outline with a single-layered epidermis and the parenchymatous cortex (Figure 4e,f and Figure 5a,b); and (ii) absence of resin canals and presence of homoxylous wood with a distinct central pith (Figure 4e,f and Figure 5c–f).

LC–ESI-MS analysis of post-mortem samples confirmed the suspicion of *T. baccata* toxicosis and allowed the detection of 20 yew-related phytochemicals (Table 1). Eighteen compounds were detected in the gastric content, nine in the liver, and only two in both kidney and fecal samples, indicating that gastric content represents the most informative biological matrix in these cases.

Among the detected compounds, several taxane-type diterpenoid alkaloids, including taxol A, taxol B, and various taxine A and B derivatives, were identified, consistent with the known toxic constituents of yew [28]. Representative total ion chromatograms obtained from gastric content, liver, kidney, and fecal samples are shown in Supplementary Figure S2, while the corresponding mass spectra of tentatively identified compounds are reported in Supplementary Figure S3.

Taxine-related compounds represented the most abundant group, including several mono-hydroxylated and mono-acetylated derivatives such as monoacetyltaxine (MAT), monohydroxymonoacetyltaxine-1 and -2 (MHMAT-1 and MHMAT-2), diacetylated derivatives such as monohydroxydiacetyltaxine-1 and -2 (MHDAT-1 and MHDAT-2), and tri-acetylated derivatives such as monohydroxytriacetyltaxine (MHTAT) and triacetyltaxine (TAT). A deacetyltaxine derivative was also detected.

All these compounds, except for MHMAT-1 and MHMAT-2, were detected exclusively in the gastric content, together with taxol A, taxol B, 10-deacetylbaccatin III (DAB) and a deacetylabeobaccatin derivative. Conversely, baccatin III was also detected in the liver, which was the only post-mortem sample in which hydroxypaclitaxel and hydroxybaccatin derivatives were observed.

Finally, gallocatechin and a triacetoxy-hydroxytaxine derivative were the only non-specific metabolites detected in both kidney and fecal samples.

## 4. Discussion

Fatal yew intoxication remains a relevant concern for animal health, despite the well-known toxicity of this plant. Nevertheless, a single, reliable diagnostic approach for the rapid identification of *Taxus* intoxication is still lacking, particularly in light of the rapid onset of clinical signs following ingestion and the high risk of sudden death.

In this study, two fatal cases involving accidental oral consumption of *T. baccata* in donkeys from the same farm are described. The cause of death, consistent with *Taxus* intoxication, was supported by anatomopathological findings, botanical examination, and toxicological analysis by LC-ESI-MS. Necropsy findings alone were not sufficient to establish the cause of death; however, the observed lesions were consistent with those reported in the literature for yew intoxication in herbivores [13,18,22]. Additionally, needle-like, flat leaves resembling those of *T. baccata* were found in the stomach of case 1.

The microscopic botanical examination of plant fragments from the gastric contents enabled the identification of leaves and twigs of European yew, even in case 2, where macroscopic identification was not possible. Indeed, the anatomical and micromorphological features used for plant identification are considered taxonomically robust and can remain preserved even after post-mortem degradation, allowing recognition in partially digested material [24]. In particular, microscopic analysis revealed the typical anatomical features of *T. baccata*, as described by Ghimire et al. [29] and Finsinger and Tinner [30], including the bifacial leaf structure with a single vascular bundle surrounded by transfusion tissue and the absence of resin canals. Another important diagnostic feature was the presence of *Taxus*-type stomata with lignified subsidiary papillose cells. The presence of 4–8 subsidiary papillose cells located above the upper lamellae and showing short polar hooks was consistent with the description reported by Finsinger and Tinner [30]. The lignification of the subsidiary cells, a typical feature of gymnosperm stomata, as described by Hu et al. [31] and Lacourse et al. [32], contributes to their preservation in biological matrices such as gastric contents.

The combination of microscopic botanical examination with pathological and toxicological findings also allowed to confirm two cases of equine intoxication by *Taxus* [22,33].

Analytical toxicology performed by LC-ESI-MS allowed the detection of 20 yew-related compounds in post-mortem samples, in agreement with previous reports in yew plant material and biological samples from intoxication cases [28,34]. These findings further support exposure to *T. baccata*, although compound identification should be considered tentative in the absence of comprehensive reference standards.

The analytical approach adopted in this study provided a rapid screening of taxane-related compounds, with a total analysis time of less than 40 min, including sample preparation. Although primarily applicable in a post-mortem context, such an approach may also be useful in acute clinical settings to support timely diagnostic decision-making. Several analytical methods based on liquid or gas chromatography coupled with mass spectrometry have been developed for the detection of taxanes and their metabolites. However, liquid chromatography remains the preferred technique for these analytes, as gas chromatography typically requires more extensive sample preparation [28]. An additional advantage of LC-based approaches lies in the availability of spectral libraries that facilitate compound assignment, although confirmation with authentic standards remains limited for several taxine derivatives due to their restricted availability [35].

In this context, the leaves (needles) of *T. baccata* represent the principal toxic organ, containing the highest concentrations of cardiotoxic alkaloids, mainly taxines A and B. While the seeds are also highly toxic, their risk is primarily associated with mastication or rupture of the seed coat, which otherwise limits toxin release during digestion. The bark and young shoots may contain variable amounts of taxines but are generally considered less relevant in acute intoxications. In contrast, the fleshy red aril is not toxic, though it may contribute indirectly to poisoning if the enclosed seed is ingested. These considerations are consistent with the findings of the present study, in which plant material compatible with yew leaves was detected in the gastric contents.

The toxicity of *T. baccata* may vary depending on developmental stage and seasonal factors, although such variations do not significantly reduce its toxic potential. Phytochemical studies indicate that juvenile plants may contain slightly lower concentrations of taxine alkaloids compared to mature individuals; however, these levels remain sufficient to cause fatal intoxication in monogastric species [6]. Seasonal fluctuations in the concentration of major taxanes, including 10-deacetylbaccatin III, paclitaxel, and baccatin III, have been reported, with higher levels typically observed during late autumn and winter [36]. Nevertheless, *Taxus* species remains highly toxic throughout the year, as taxines are chemically stable and retain their activity even in dried plant material [37].

In the present cases, seasonal variation is unlikely to have influenced the outcome, given the high susceptibility of monogastric animals to acute toxicosis. Indeed, ingestion of *T. baccata* leaves at doses as low as 0.1% of body weight is considered lethal in these species [38]. This high sensitivity, combined with the rapid onset of cardiotoxic effects, supports the diagnostic relevance of detecting *T. baccata* in suspected cases of intoxication.

Although the present study focused on *T. baccata*, the only *Taxus* species documented in the study area, it is acknowledged that, in other geographical contexts, the presence of morphologically similar species (e.g., *T. brevifolia*) may require additional identification tools. In such cases, anatomical and micromorphological analysis may be complemented by DNA-based approaches methods. Recent studies have described molecular markers capable of distinguishing closely related *Taxus* species, including *T. baccata*, highlighting the potential of PCR-based approaches for species-level identification [39].

Although the absence of additional biological matrices such as blood, urine, or bile represents a limitation, the detection of yew residues in gastric contents, together with the identification of taxane-related compounds and liver metabolites such as hydroxypaclitaxel and hydroxybaccatin derivatives, supports systemic exposure following ingestion of *T. baccata*.

Taxane metabolism is primarily hepatic, with minimal renal excretion (<5% in urine) [40]. These compounds are metabolized by cytochrome P450 enzymes and eliminated via biliary excretion into the feces. The limited detection of metabolites in fecal samples in the present cases is consistent with a rapid and fatal course of intoxication, which may also explain the associated hepatic lesions observed at histopathological examination.

## 5. Conclusions

These cases are consistent with a hyperacute course of intoxication, and the multidisciplinary investigations conducted to assess the cause of death supported the conclusion that the animals died following ingestion of *T. baccata*.

The diagnostic outcome was facilitated by the integration of anamnestic information provided by the owners, the detection of yew plant material within the gastric contents, and the application of complementary analytical approaches, including botanical identification and LC-ESI-MS toxicological analysis.

## Figures and Tables

**Table 1:** LC-ESI-MS analysis of necropsy samples collected from two donkeys (case 1 and 2) following suspected ingestion of toxic substances.

n.	Compound Name	MW	[H + H]^+^	Gastric Content	Liver	Kidneys	Feces
1	Paclitaxel (Taxol A) *	854	855	+	−	−	−
2	Hydroxypaclitaxel	870	871	−	+	−	−
3	Monohydroxymonoacetyltaxine-1 (MHMAT-1)	583	584	+	+	−	−
4	Monohydroxymonoacetyltaxine-2 (MHMAT-2)	583	584	+	+	−	−
5	10-Deacetylbaccatin III (DAB) *	547	548	+	−	−	−
6	Monoacetyltaxine (MAT)	567	568	+	−	−	−
7	Baccatin III (BAC III)	587	588	+	+	−	−
8	Monohydroxydiacetyltaxine (MHDAT-1)	625	626	+	−	−	−
9	Cephalomannine (Taxol B)	832	833	+	−	−	−
10	Deacetyltaxine derivative	601	602	+	−	−	−
11	Deacetylabeobaccatin derivative	568	569	+	−	−	−
12	Monohydroxydiacetyltaxine-2 (MHDAT-2)	625	626	+	−	−	−
13	Hydroxybaccatin derivative	603	604	−	+	−	−
14	Monohydroxytriacetyltaxine (MHTAT)	667	668	+	−	−	−
15	Hydroxytriacetyltaxine derivative	653	654	+	−	−	−
16	Triacetyltaxine (TAT)	651	652	+	−	−	−
17	Kaempferol malonyl-hexoside	534	535	+	+	−	−
18	Quercetin diglucoside	626	627	+	+	−	−
19	Gallocatechin	306	307	+	+	+	+
20	Triacetoxy-hydroxytaxine derivative	639	640	+	+	+	+

* Confirmed by comparison with reference standards; ‘+’ indicates the presence of the compound, whereas ‘−’ indicates that the compound was not detected.

## Data Availability

The original contributions presented in this study are included in the article/Supplementary Material. Further inquiries can be directed to the corresponding author.

## Supplementary Materials

The following supporting information can be downloaded at: https://www.mdpi.com/article/10.3390/toxics14040294/s1. Figure S1: (a) Edge of the donkey grazing area, where a European yew tree (*T. baccata*) is visible, whose branches are close to the fence wall (white arrows); photo courtesy of the owner of the animals; (b) High magnification detail of the opposed needles on horizontally protruding twigs of a *T. baccata* tree, for comparison. Figure S2: Representative Total Ion Current (TIC) chromatograms obtained from gastric content (A), liver (B), kidney (C), and fecal (D) samples. Compound numbering corresponds to that reported in Table 1 of the main text. Figure S3: Mass spectra of tentatively identified compounds detected at the following retention times: 3.7 min (compound **1**), 4.2 min (compound **2**), 5.0 min (compound **3**), 5.7 min (compound **4**), 7.8 min (compound **5**), 8.2 min (compound **6**), 8.4 min (compound **7**), 8.7 min (compound **8**), 8.9 min (compound **9**), 9.1 min (compound **10**), 9.3 min (compound **11**), 9.6 min (compound **12**), 9.8 min (compound **13**), 10.0 min (compound **14**), 10.3 min (compound **15**), 10.6 min (compound **16**), 11.0 min (compound **17**), 13.7 min (compound **18**), 17.2 min (compound **19**), and 19.3 min (compound **20**). Compound numbering corresponds to that reported in Table 1 of the main text.
