# Supplementary material for: Fatal Intoxication of European Yew (Taxus baccata L.) in Two Donkeys in North-Eastern Italy: A Case Report
Source: Toxics. 2026 Mar 28;14(4):294. doi: 10.3390/toxics14040294 (PMC13119995; doi:10.3390/toxics14040294)
Supplement: Supplementary file 1 [file toxics-14-00294-s001.zip › Supplementary Material Figure 1.pdf]

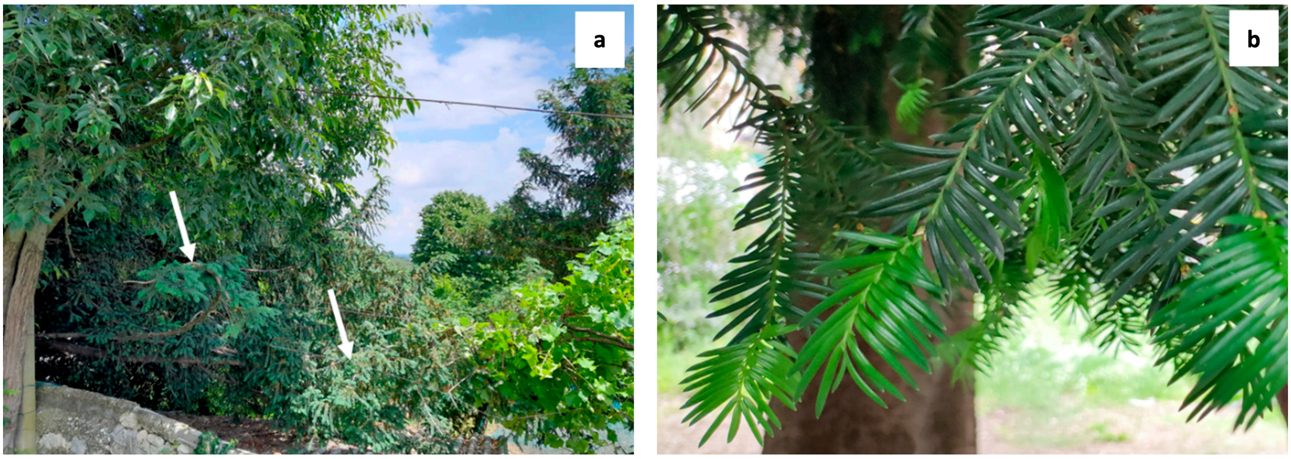

Figure S1. (a) Edge of the donkey grazing area, where European yew tree (*T. baccata*) is visible, whose branches are close to the fence wall (white arrows); photo courtesy of the owner of the animals; (b) High magnification detail of the opposed needles on horizontally protruding twigs of a *T. baccata* tree, for comparison.
