# Supplementary material for: Fatal Intoxication of European Yew (Taxus baccata L.) in Two Donkeys in North-Eastern Italy: A Case Report
Source: Toxics. 2026 Mar 28;14(4):294. doi: 10.3390/toxics14040294 (PMC13119995; doi:10.3390/toxics14040294)
Supplement: Supplementary file 1 [file toxics-14-00294-s001.zip › Supplementary material_Figure S2_revised.pdf]

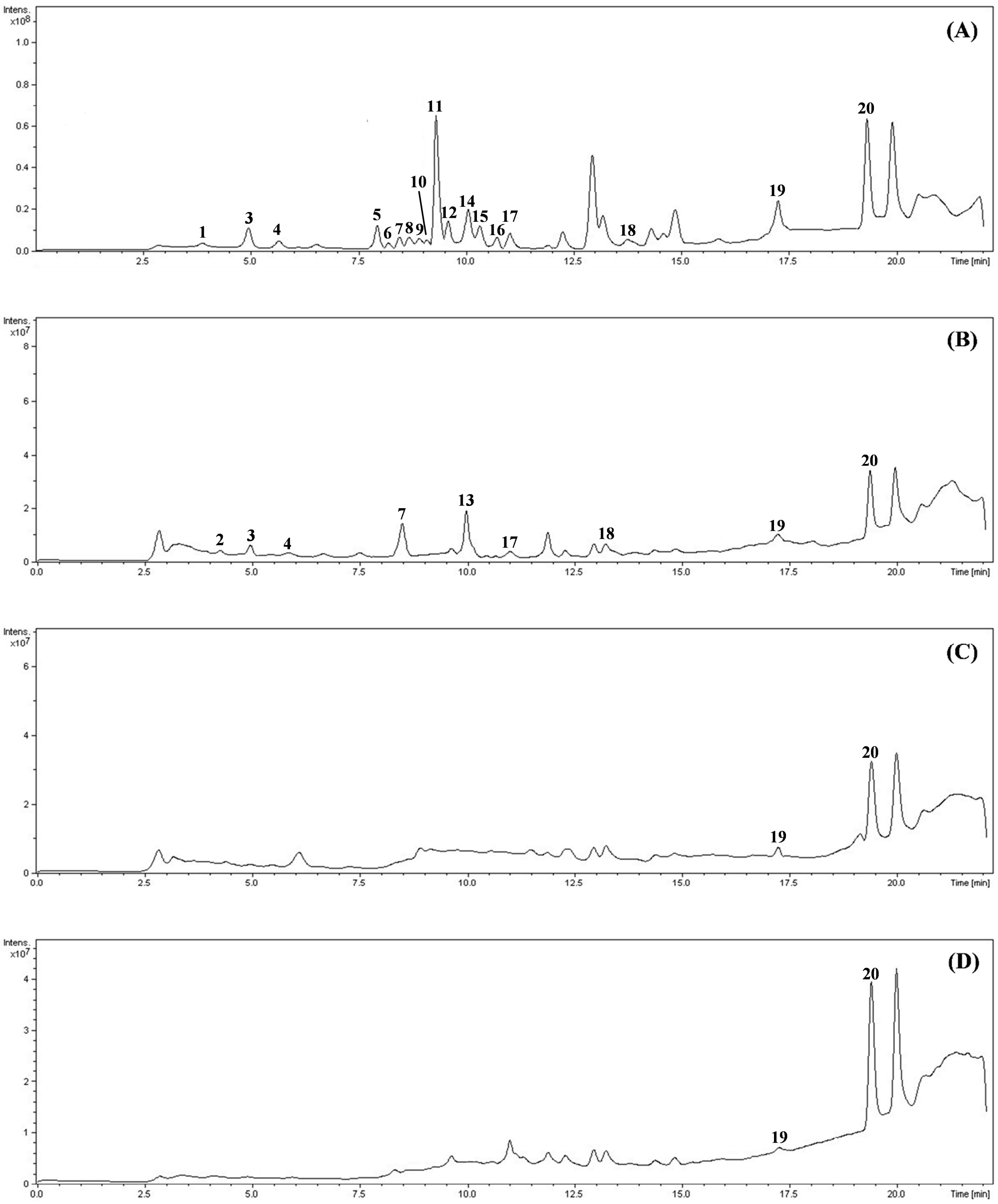

Figure S2. Representative Total Ion Current (TIC) chromatograms obtained from gastric content (A), liver (B), kidney (C), and faeces (D) samples. Compound numbering corresponds to that reported in Table 1 of the main text.
