# Supplementary material for: Fatal Intoxication of European Yew (Taxus baccata L.) in Two Donkeys in North-Eastern Italy: A Case Report
Source: Toxics. 2026 Mar 28;14(4):294. doi: 10.3390/toxics14040294 (PMC13119995; doi:10.3390/toxics14040294)
Supplement: Supplementary file 1 [file toxics-14-00294-s001.zip › Supplementary material_Figure S3.pdf]

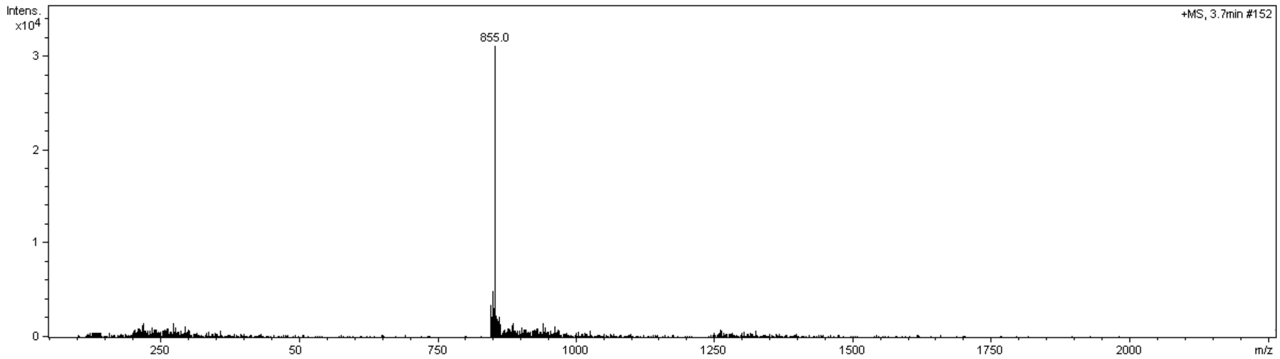
Intens.
x10^4
+MS, 3.7min #152
855.0
3
2
1
0
250
50
750
1000
1250
1500
1750
2000
m/z

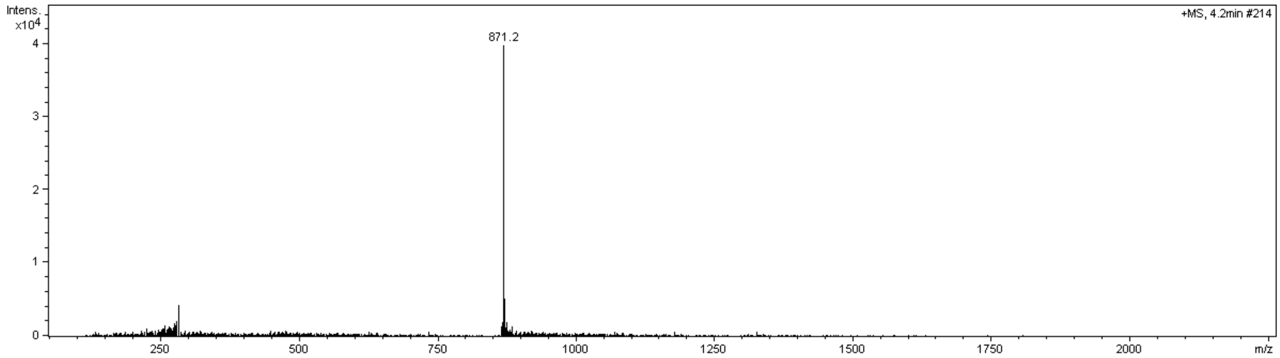
Intens.
x10^4
+MS, 4.2min #214
871.2
4
3
2
1
0
250
500
750
1000
1250
1500
1750
2000
m/z

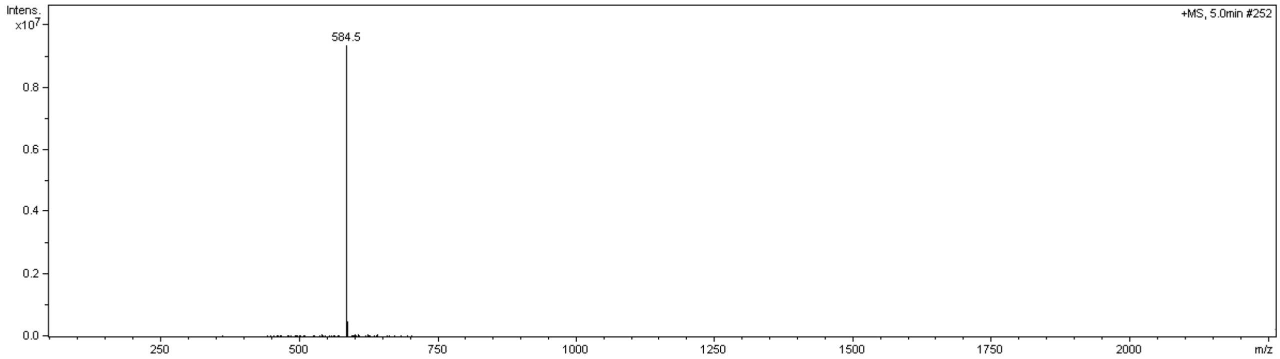
Intens.
x10^7
+MS, 5.0min #252
584.5
0.8
0.6
0.4
0.2
0.0
250
500
750
1000
1250
1500
1750
2000
m/z

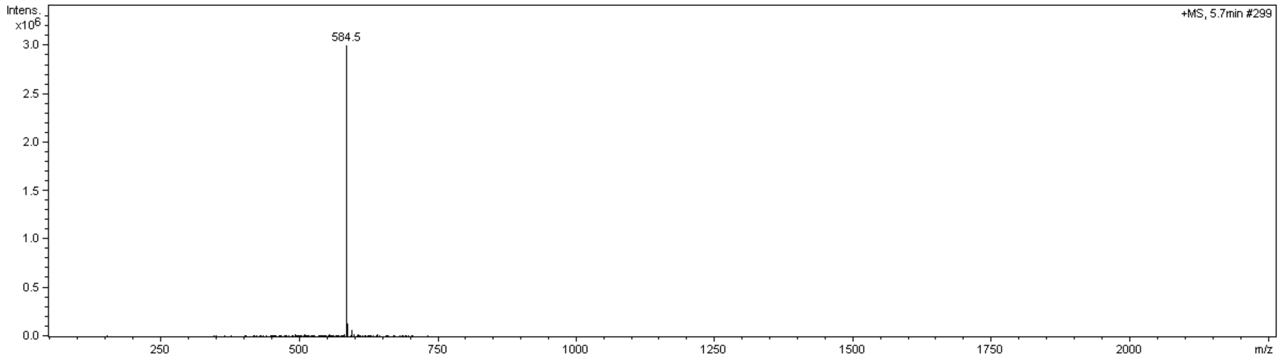
Intens.
x10^6
+MS, 5.7min #299
584.5
3.0
2.5
2.0
1.5
1.0
0.5
0.0
250
500
750
1000
1250
1500
1750
2000
m/z

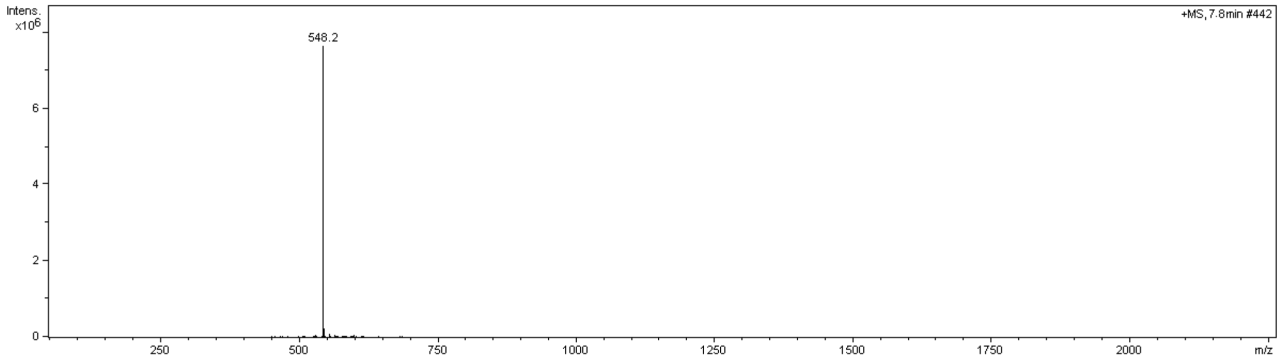
Intens.
x10^6
+MS, 7.8min #442
548.2
0
2
4
6
250
500
750
1000
1250
1500
1750
2000
m/z

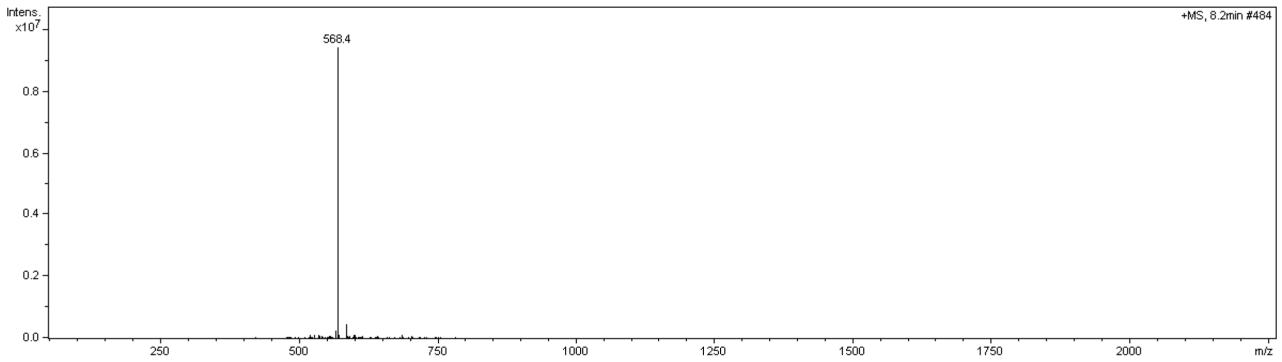
Intens.
x10^7
+MS, 8.2min #484
568.4
0.0
0.2
0.4
0.6
0.8
250
500
750
1000
1250
1500
1750
2000
m/z

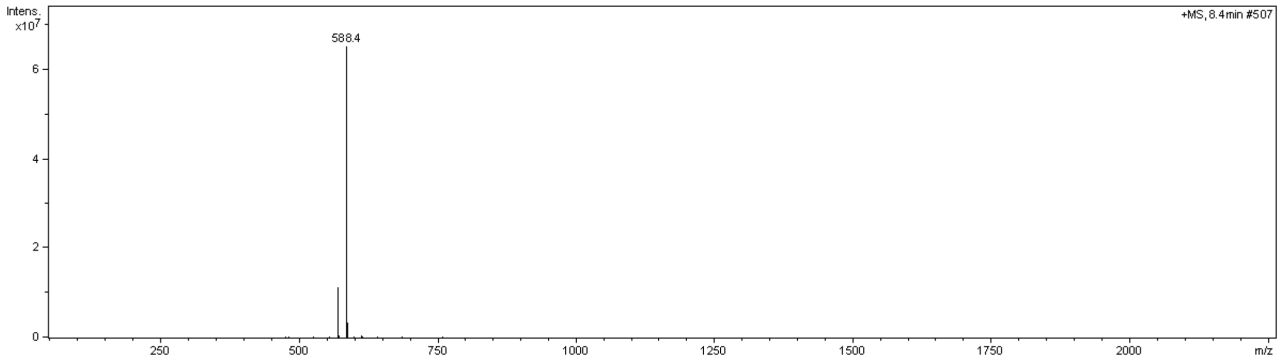
Intens.
x10^7
+MS, 8.4min #507
588.4
0
2
4
6
250
500
750
1000
1250
1500
1750
2000
m/z

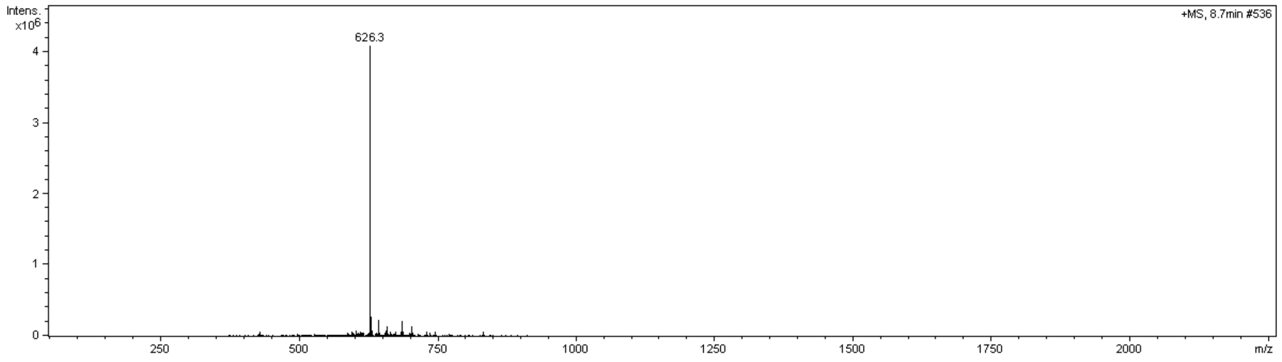
Intens.
x10^6
+MS, 8.7min #536
626.3
0
1
2
3
4
250
500
750
1000
1250
1500
1750
2000
m/z

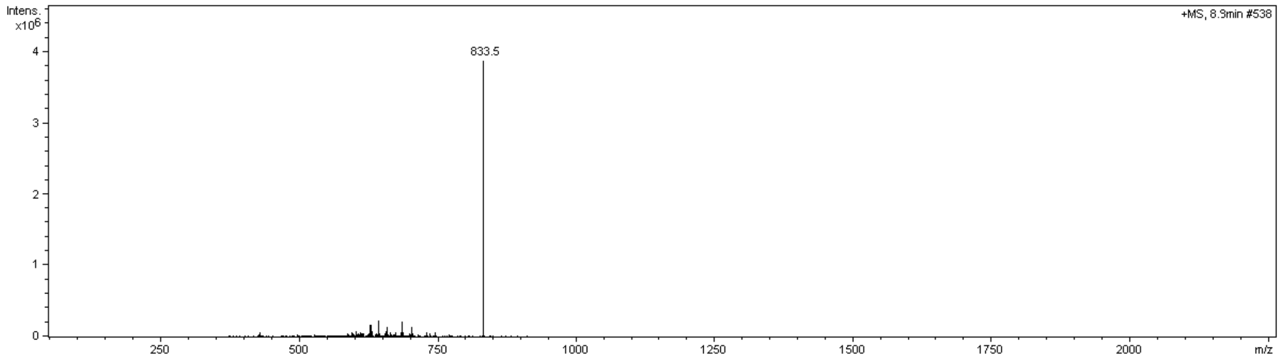
Intens.
x10^6
+MS, 8.9min #538
833.5
m/z

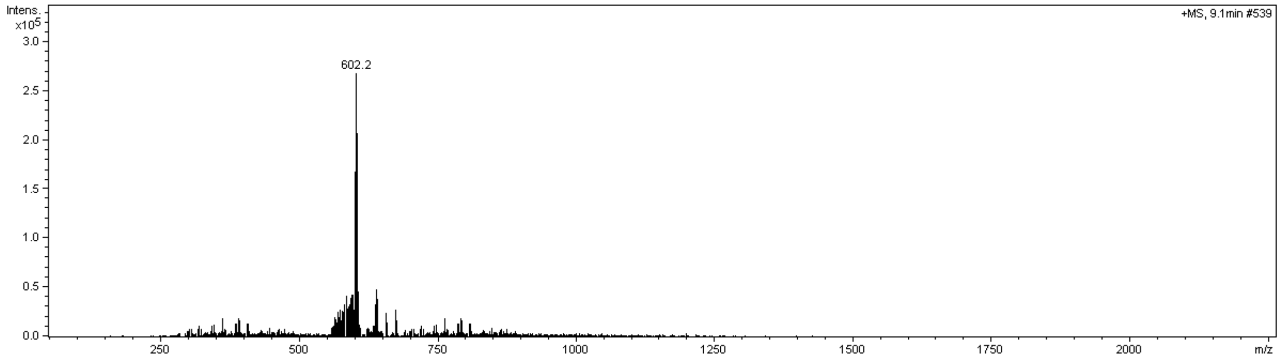
Intens.
x10^5
+MS, 9.1min #539
602.2
m/z

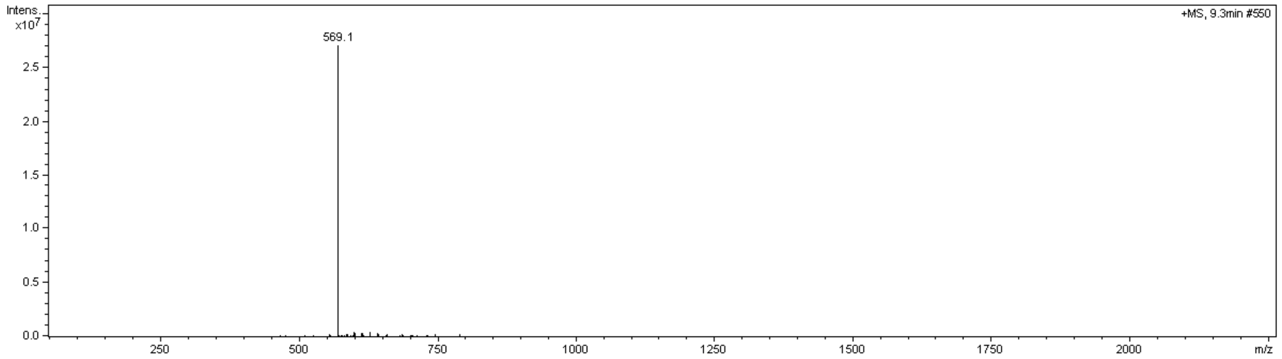
Intens.
x10^7
+MS, 9.3min #550
569.1
m/z

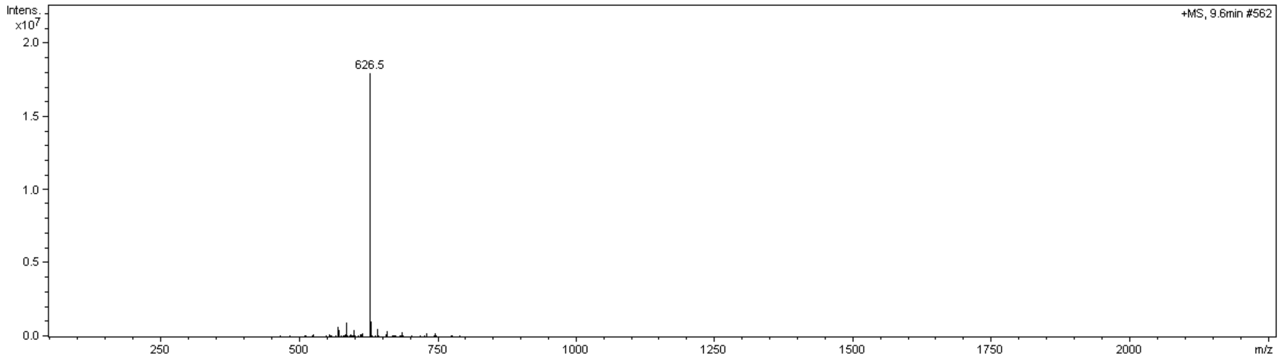
Intens.
x10^7
+MS, 9.6min #562
626.5
m/z

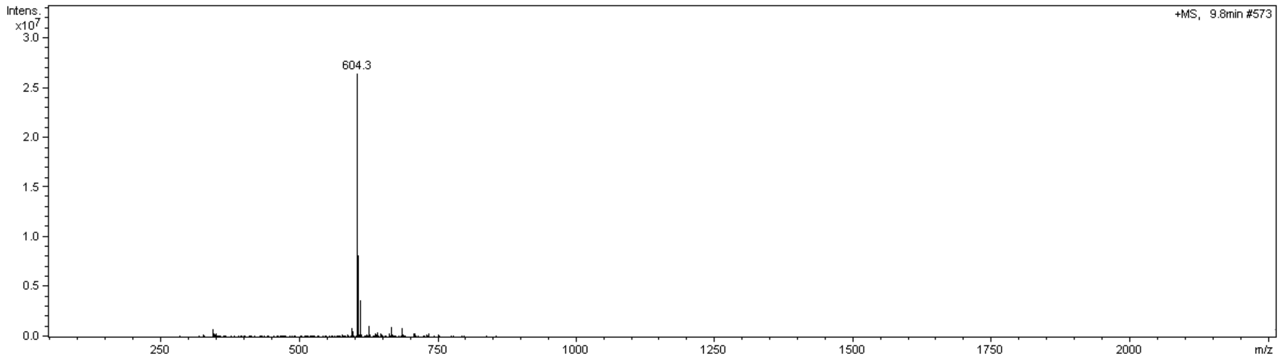
Intens.
x10^7
+MS, 9.8min #573
604.3
m/z

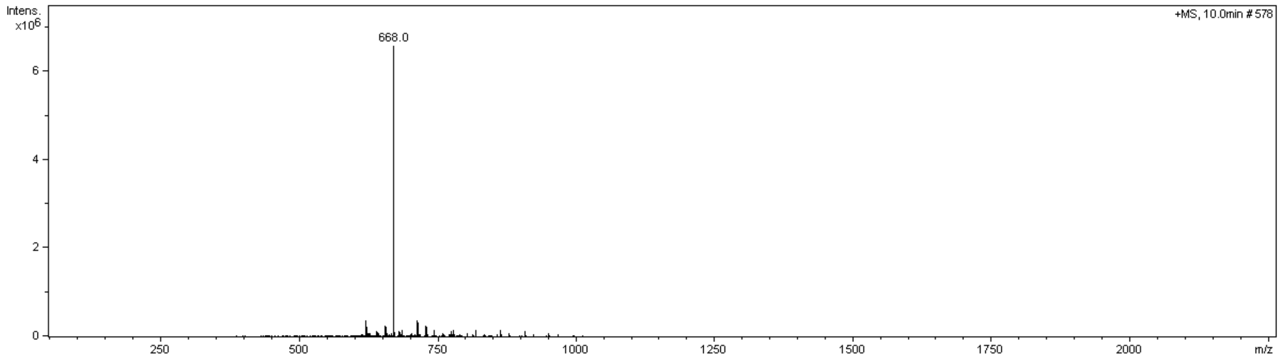
Intens.
x10^6
+MS, 10.0min #578
668.0
m/z

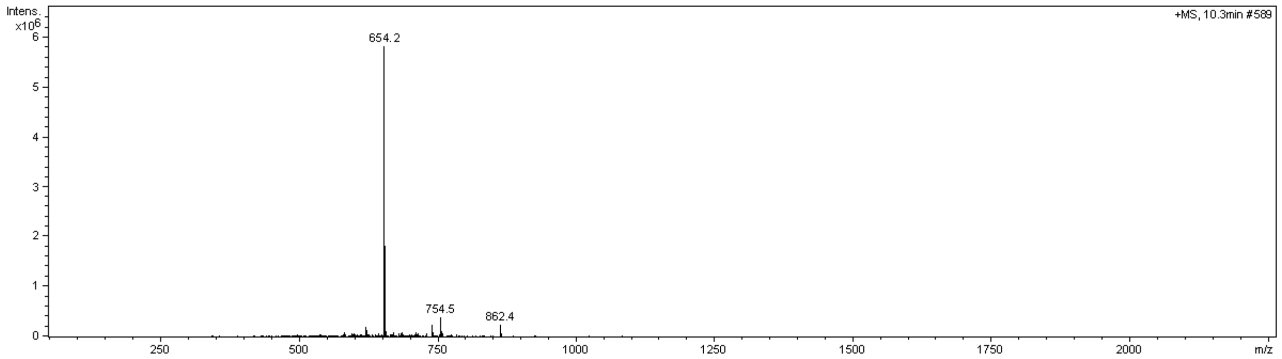
Intens.
x10^6
+MS, 10.3min #589
654.2
754.5
862.4
m/z

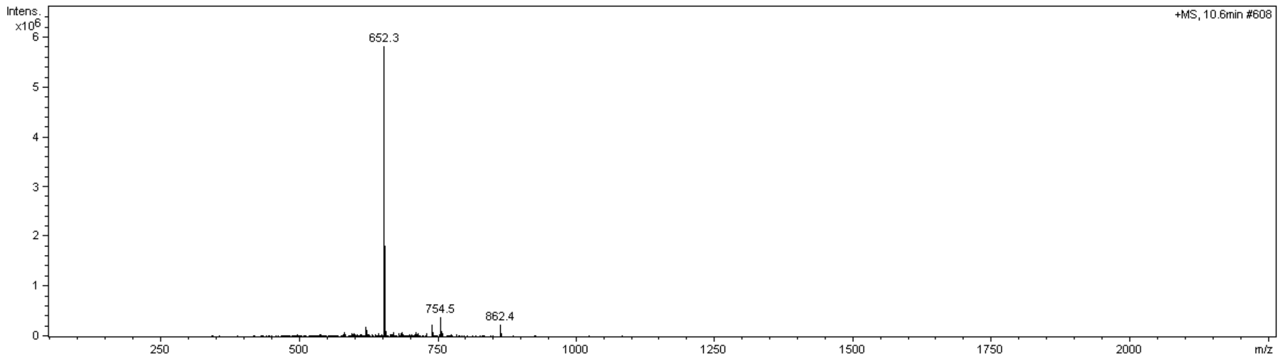
Intens.
x10^6
+MS, 10.6min #608
652.3
754.5
862.4
m/z

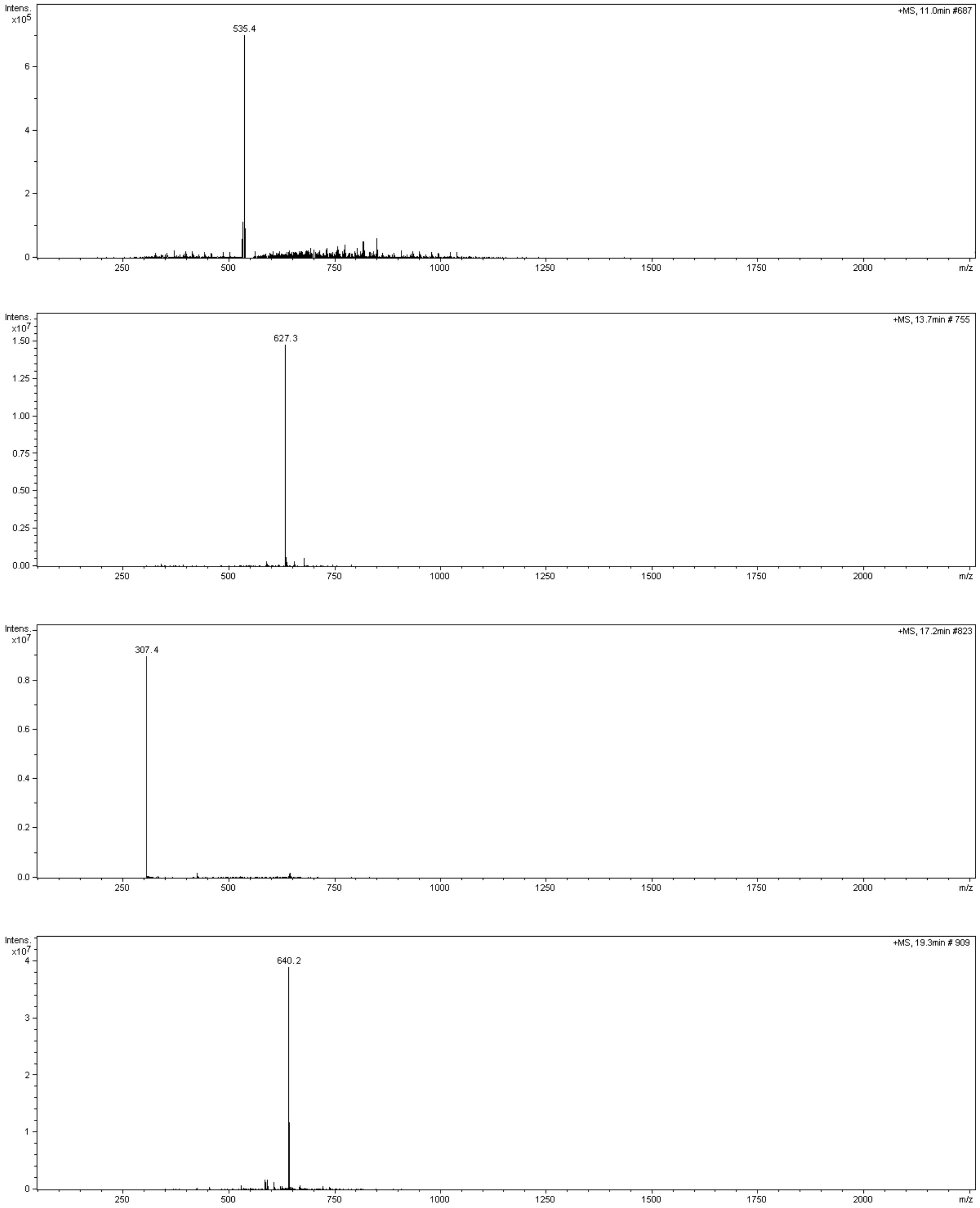

Figure S3. Mass spectra of tentatively identified compounds detected at the following retention times: 3.7 min (compound 1), 4.2 min (compound 2), 5.0 min (compound 3), 5.7 min (compound 4), 7.8 min (compound 5), 8.2 min (compound 6), 8.4 min (compound 7), 8.7 min (compound 8), 8.9 min (compound 9), 9.1 min (compound 10), 9.3 min (compound 11), 9.6 min (compound 12), 9.8 min (compound 13), 10.0 min (compound 14), 10.3 min (compound 15), 10.6 min (compound 16), 11.0 min (compound 17), 13.7 min (compound 18), 17.2 min (compound 19), and 19.3 min (compound 20). Compound numbering corresponds to that reported in Table 1 of the main text.
